# Supplementary material for: HIV replication and tuberculosis risk among people living with HIV in Europe: A multicohort analysis, 1983–2015
Source: PLoS One. 2024 Oct 25;19(10):e0312035. doi: 10.1371/journal.pone.0312035 (PMC11508122; doi:10.1371/journal.pone.0312035)
Supplement: S1 Table — (PDF) [file pone.0312035.s001.pdf]

S1 Table. Breakdown of participants in the study by cohort.

Cohort	N	%	
AHIVCOS	4,456	1.6	Austria
AMACS	6,475	2.4	Greece
AQUITAINE	6,516	2.4	France
ATHENA	20,116	7.4	The Netherlands
Bonn	1,473	0.5	Germany
ClinSurv	20,409	7.5	Germany
CO-RISMD	2,960	1.1	Spain
Cologne	2,481	0.9	Germany
COPILOTE	1,209	0.4	France
DHK	4,752	1.7	Denmark
EuroSIDA [1]	12,086	4.4	Eastern Europe
FHDH	80,983	29.7	France
HEPAVIH	1,106	0.4	France
HSR San Raffaele	2,985	1.1	Italy
ICC	678	0.2	Italy
ICONA	10,473	3.8	Italy
Modena	527	0.2	Italy
PISCIS	12,339	4.5	Spain
PRIMO	1,728	0.6	France
SEROCO	859	0.3	France
SHCS	7,936	2.9	Switzerland
StPierre	4,464	1.6	Belgium
Sweden Infcare	6,013	2.2	Sweden
UK CHIC	43,139	15.8	United Kingdom
VACH	16,385	6	Spain
**Total**	**272,548**	**100**	

[1] EuroSIDA is a prospective observational HIV cohort study covering all European regions (including East Central and Eastern Europe), Israel, and Argentina. Included cohorts were from Belarus, Croatia, Czech Republic, Estonia, Hungary, Latvia, Lithuania, Romania, Russian Federation, Serbia, Slovakia, Slovenia, and Ukraine.
