# Supplementary figures and images for: HIV replication and tuberculosis risk among people living with HIV in Europe: A multicohort analysis, 1983–2015
Source: PLoS One. 2024 Oct 25;19(10):e0312035. doi: 10.1371/journal.pone.0312035 (PMC11508122; doi:10.1371/journal.pone.0312035)

**S1 Figure. Selection of the study population.**

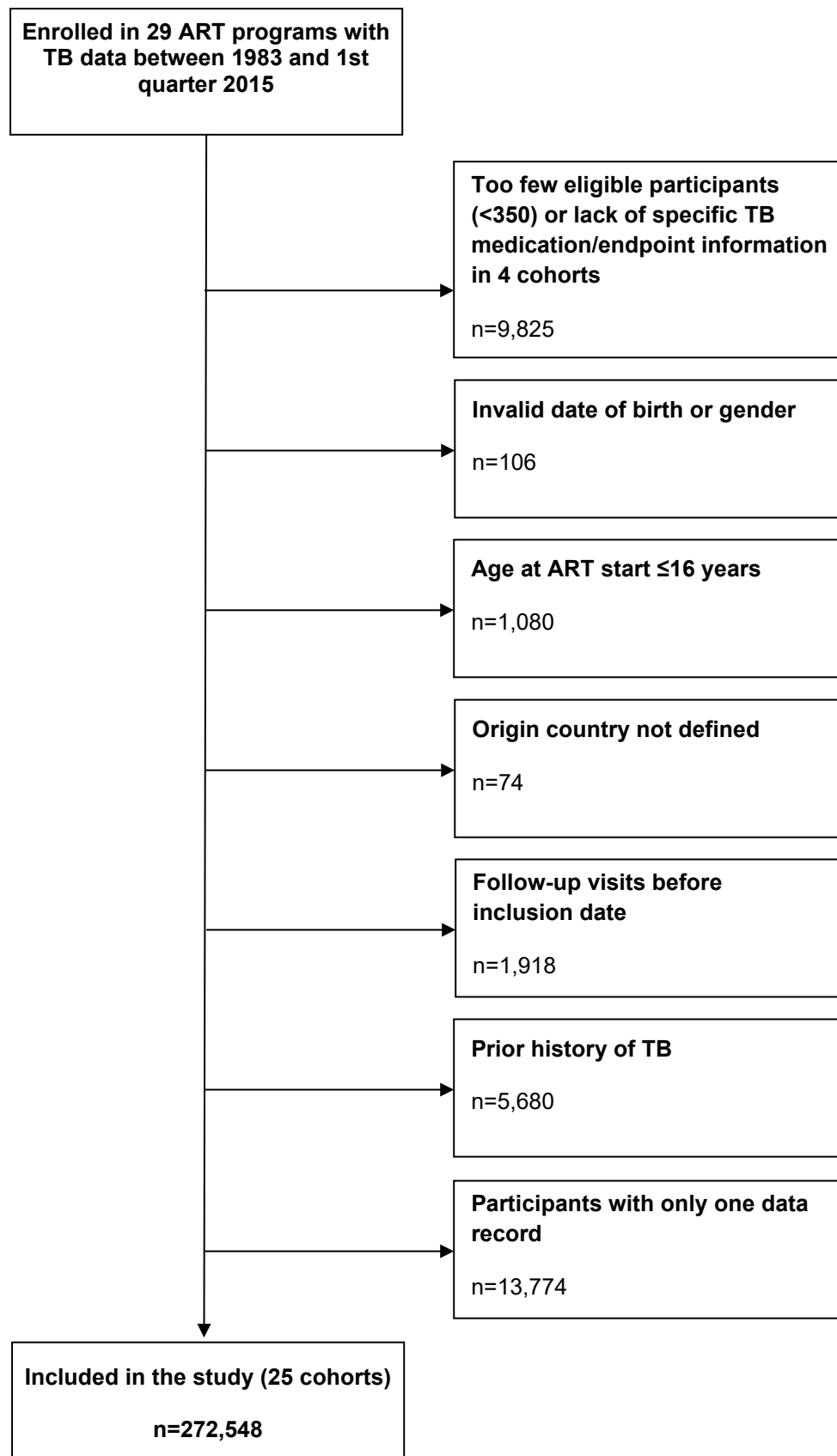

ART, antiretroviral therapy; TB, tuberculosis

Supplement: S1 Fig — (PDF) [file pone.0312035.s002.pdf]
