# Supplementary material for: HIV replication and tuberculosis risk among people living with HIV in Europe: A multicohort analysis, 1983–2015
Source: PLoS One. 2024 Oct 25;19(10):e0312035. doi: 10.1371/journal.pone.0312035 (PMC11508122; doi:10.1371/journal.pone.0312035)

S2 Fig. TB incidence rate per 1,000 person-years for a reference population, based on time-updated measurements and stratified by CD4 counts (reference: 35 years of age, male, heterosexual, on ART). A: All regions are shown unless the region was unknown. B: Only sub-Saharan Africa, Asia, and Europe are shown for clarity.

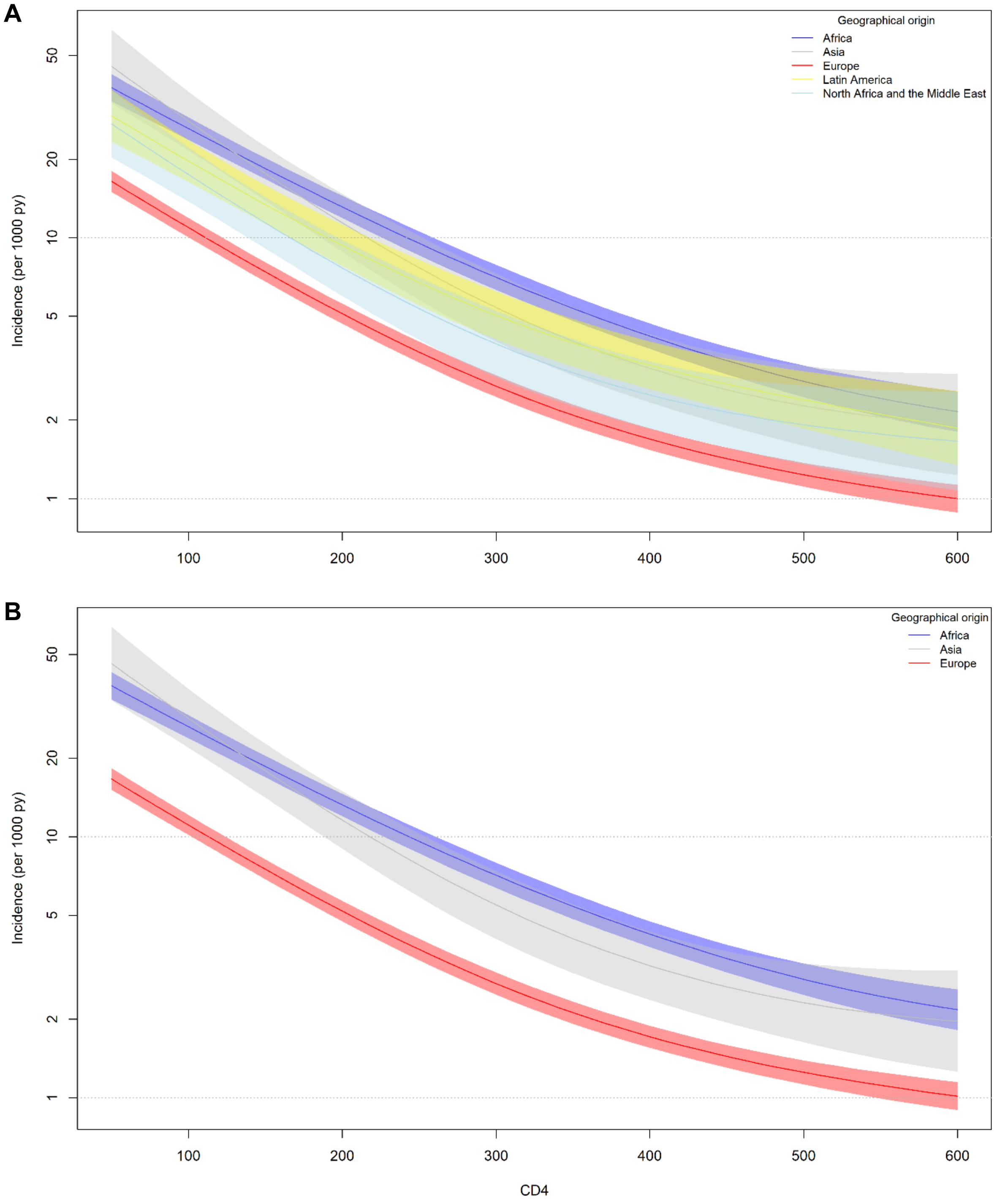

Supplement: S2 Fig — A: All regions are shown unless the region was unknown. B: Only sub-Saharan Africa, Asia, and Europe are shown for clarity. (PDF) [file pone.0312035.s003.pdf]
