# Supplementary material for: HIV replication and tuberculosis risk among people living with HIV in Europe: A multicohort analysis, 1983–2015
Source: PLoS One. 2024 Oct 25;19(10):e0312035. doi: 10.1371/journal.pone.0312035 (PMC11508122; doi:10.1371/journal.pone.0312035)
Supplement: Attachment — Submitted filename: Response_TB_RNA_COHERE_PLoSONE_rev1_final.docx [file pone.0312035.s004.docx]

**RESPONSES TO EDITORIAL AND REVIEWER’S COMMENTS**

**Manuscript ID: PONE-D-24-10899**

**Manuscript title: HIV replication and tuberculosis risk among people living with HIV in Europe: a multicohort analysis,** **1983-2015**

**Dear Editor,**

**Thank you for carefully reviewing our manuscript. We appreciate the overall positive and helpful feedback and welcome the opportunity to submit a revised version of our manuscript.**

**Below we provide a point-by-point response to each of the comments.**

**Changes that we have incorporated during the revision process are highlighted in blue in the revised manuscript.**

**Yours sincerely,**

**Lukas Fenner, on behalf of the co-authors**

**Reviewer #1**

The manuscript provides an excellent analysis of HIV cohort spanning over many decades from European countries. Below are some of the comments to authors.

Author response: Many thanks for the positive feedback.

1. There are several typos that require attention i.e line 106, line 124.

Author response: Thank you. This has been corrected.

2. Data availability is provided under two websites. However, the COHERE website is inaccessible or does not work.

Author response: Because the COHERE website is no longer actively maintained and updated, we did not include a website reference in the manuscript. Our data availability statement is as follows (p. 8):

*“Data used for the analysis will generally not be publicly available but can be made available based on the approval by the chair of the executive committee of COHERE (Stephane De Wit; refer to author list).”*

3. Ethics statement is provided but does not include how data was protected and confidentiality maintained. A follow-up statement would be helpful.

Author response: In COHERE, ethical requirements fall within the individual cohort’s ethics. All COHERE cohorts follow local ethical standards. Please also refer to the COHERE cohort profile <https://doi.org/10.1093/ije/dyw211>.

To address the Reviewer’s concern, we revised the ethics statement as follows and added the COHERE cohort profile (p. 5):

*“**Ethical approval was applied for and granted for the research from the appropriate body in the host country (local ethics committees or institutional review boards) of the cohort contributing the data to COHERE [20]. All cohorts participating in COHERE adhere to local ethical, data management and confidentiality standards.”*

4. It is not clear how TB diagnosis was done and/or a local diagnostic criteria could be provided.

Author response: In COHERE, data were primarily extracted from medical records in the context of routine care and follow local diagnostic criteria. Unfortunately, we cannot provide detailed local diagnostic criteria for each of the cohorts. We have clarified this in the Methods section as follows (p. 5):

*“The diagnosis of TB was made by the treating clinicians according to local diagnostic criteria.”*

5. History of BCG vaccination is important and information on its availability or implications for the study is key. Some countries use it as part of their TB prevention strategy.

Author response: This is an interesting point. In general, indications for BCG immunisation vary considerably in Europe (https://doi.org/10.1016/j.vaccine.2015.06.097). Since COHERE did not collect information on the history of BCG, we cannot provide further details.

6. A general comment on the availability of ART would be useful since the cohort started in 1983. Which year was ART made available to the participants?

Author response: Thank you for pointing this out. ART access generally started with zidovudine monotherapy in 1987. In Europe, , and widespread access to effective combination antiretroviral therapy started in 1996 and has dramatically reduced the number of deaths among people living with HIV (<https://doi.org/10.1016/S0140-6736(03)13802-0>).

We therefore added the following sentence (p. 6):

*“In Europe, widespread access to effective combination ART began in 1996 and has dramatically reduced the number of deaths among people living with HIV [20,24].”*

7. Other risk factors identified could be reported i.e alcohol, nutrition, smoking, non-adherence etc.

Author response: We agree with the Reviewer that other risk factors for TB are important. Unfortunately, the COHERE database does not include this information.

We have added this limitation in the Discussion section (p. 7):

“*Other limitations include the limited number of people with HIV followed in Eastern Europe, which has a different TB epidemiology [37], and that there may be residual confounding (homelessness, instable social conditions, alcohol use) that was not captured in our dataset.*”

8. Future implications of TPT based on HIV viral load could be added to the recommendations. Some previous recommendations were based on CD4 count.

Author response: Thank you for your suggestion. In countries with a high TB/HIV burden, WHO recommends TPT for adults, adolescents, children, and infants with HIV, regardless of the degree of immunosuppression and even when latent TB infection testing is not available. People with HIV at high risk for TB in Europe, those with higher levels of ongoing HIV replication as identified in our study, may particularly benefit from the administration of TPT.

We state in the Discussion (p. 7):

*“People with HIV who have detectable HIV viral loads at any time are at increased risk of active TB by reactivation of latent TB, reinfection, or rapid progression after recent infection, regardless of their CD4 cell counts. Therefore, in addition to early initiation of ART or optimization of ART, these individuals may particularly benefit from the administration of TPT to reduce the risk of TB in persons with latent TB, especially in migrant populations originating from TB-endemic countries and possibly higher exposure to TB. […] In any case, people at high risk identified in our study should be considered for latent TB screening and TPT if active TB has been excluded and should be closely monitored during HIV care to reduce the risk of treatment interruptions and eventually ensure early diagnosis of TB.”*

**Reviewer #2**

TB is still the leading cause of death among PLHIV even in countries that have achieved optimal ART roll out. The authors show the impact of suboptimal HIV control on TB incidence and that this relationship between detectable viral load and TB incidence is maintained at all CD4 strata.
These findings are important for countries with large TB and HIV burden and further emphasize the need for frequent viral load monitoring among PLHIV started on ART and the importance of additional TB preventive measures e.g., TB preventive therapy among persons living with HIV.

Author response: Many thanks for the overall positive feedback.

**Journal requirements**

1. Please ensure that your manuscript meets PLOS ONE's style requirements, including those for file naming.

Author response: We modified the manuscript according the journal requirements, including title page, legends, in-text citations, and references. We also corrected the affiliations. We also updated the Acknowledgement paragraph to comply with COHERE’s policy.

2. We noticed you have some minor occurrence of overlapping text with the following previous publication(s), which needs to be addressed: <https://doi.org/10.7448/IAS.20.1.21327>. In your revision ensure you cite all your sources (including your own works), and quote or rephrase any duplicated text outside the methods section. Further consideration is dependent on these concerns being addressed.

Author response: We could not find relevant overlapping text with our previous work (Atkinson et al., JIAS 2017). However, we ensured that relevant text from our previous work is cited accordingly. Please let us know which text you think is still overlapping and is relevant in any way.

3. Please include captions for your Supporting Information files at the end of your manuscript, and update any in-text citations to match accordingly.

Author response: Many thanks. This was revised accordingly.

4. Please review your reference list to ensure that it is complete and correct. If you have cited papers that have been retracted, please include the rationale for doing so in the manuscript text, or remove these references and replace them with relevant current references. Any changes to the reference list should be mentioned in the rebuttal letter that accompanies your revised manuscript. If you need to cite a retracted article, indicate the article’s retracted status in the References list and also include a citation and full reference for the retraction notice.

Author response: We carefully reviewed the references and changed the reference style to meet PLOS ONE’s style requirements.
